# Glucose ingestion before and after resistance training sessions does not augment ribosome biogenesis in healthy moderately trained young adults

**DOI:** 10.1007/s00421-024-05446-x

**Published:** 2024-03-08

**Authors:** Kristian Lian, Daniel Hammarström, Håvard Hamarsland, Knut Sindre Mølmen, Sara Christine Moen, Stian Ellefsen

**Affiliations:** Section for Health and Exercise Physiology, Department of Public Health and Sport Sciences, Inland Norway University of Applied Sciences, Lillehammer, Norway

**Keywords:** Glucose, Hypertrophy, Resistance training, Ribosome, Skeletal muscle

## Abstract

**Purpose:**

Resistance training-induced skeletal muscle hypertrophy seems to depend on ribosome biogenesis and content. High glucose treatment may augment ribosome biogenesis through potentiating resistance training-induced adaptations. This was investigated with total RNA and ribosomal RNA abundances as main outcomes, with relevant transcriptional/translational regulators (c-Myc/UBF/rpS6) as a secondary outcome.

**Methods:**

Sixteen healthy, moderately trained individuals [male/female, *n* = 9/7; age, 24.1 (3.3)] participated in a within-participant crossover trial with unilateral resistance training (leg press and knee extension, 3 sets of 10 repetitions maximum) and pre- and post-exercise ingestion of either glucose (3 × 30 g, 90 g total) or placebo supplements (Stevia rebaudiana, 3 × 0.3 g, 0.9 g total), together with protein (2 × 25 g, 50 g total), on alternating days for 12 days. Six morning resistance exercise sessions were conducted per condition, and the sessions were performed in an otherwise fasted state. Micro-biopsies were sampled from m. vastus lateralis before and after the intervention.

**Results:**

Glucose ingestion did not have beneficial effects on resistance training-induced increases of ribosomal content (mean difference 7.6% [− 7.2, 24.9], *p* = 0.34; ribosomal RNA, 47S/18S/28S/5.8S/5S, range 7.6–37.9%, *p* = 0.40–0.98) or levels of relevant transcriptional or translational regulators (c-MYK/UBF/rpS6, *p* = 0.094–0.292). Of note, both baseline and trained state data of total RNA showed a linear relationship with UBF; a ∼14% increase in total RNA corresponded to 1 SD unit increase in UBF (*p* = 0.003).

**Conclusion:**

Glucose ingestion before and after resistance training sessions did not augment ribosomal RNA accumulation during twelve days of heavy-load resistance training in moderately trained young adults.

## Introduction

Responses to systematic resistance training (RT) vary widely between individuals, with as much as 10–15% showing impaired skeletal muscle growth in response to standardised training interventions (Thalacker-Mercer et al. 2013; Mann et al. 2014; Álvarez et al. 2018). Genetic predisposition may explain some of this variation (Thalacker-Mercer et al. 2013), but in general, the internal physiological milieu seems to be favourably shaped for muscle growth in response to different types of nutrient intake and exercise training (Thalacker-Mercer et al. 2013; Tanaka and Tsuneoka 2018; Figueiredo et al. 2021). Indeed, exercise training and nutrients impact the ability to synthesise ribosomes, which in turn is demonstrated to be connected to the magnitude of RT-induced responses (Kusnadi et al. 2015; Tanaka and Tsuneoka 2018; Hammarström et al. 2020). For instance, increasing training volume generally induces greater ribosome biogenesis and is associated with greater benefits of RT in terms of gains in skeletal muscle mass and -strength (Krieger 2009; Schoenfeld et al. 2017; Hammarström et al. 2020). Still, as evident from Hammarström et al. (2020), not all participants experience increased muscle mass and improved muscle strength with increasing training volume. Therefore, means other than modification of RT variables seem necessary to optimise individual responses to RT, for instance, nutritional adjuvants.

Nutritional supplements such as protein and creatine are frequently advocated as means to optimise RT adaptations (Cermak et al. 2012; Lanhers et al. 2015, 2017; Morton et al. 2018). The efficacy of other nutritional adjuvants such as glucose remains equivocal (Tezze et al. 2023). This is surprising given that glucose is the preferred energy substrate of the contracting skeletal muscle during strenuous exercise and a major energy supplier to cells via adenosine triphosphate (ATP) synthesis (Mul et al. 2015; Tanaka and Tsuneoka 2018). Furthermore, energy availability is a decisive factor in the de novo synthesis of ribosomes (Moss et al. 2007; Kusnadi et al. 2015; Tanaka and Tsuneoka 2018) which in turn seems to determine muscle growth by increasing the muscle’s translational capacity (Stec et al. 2016; Tanaka and Tsuneoka 2018; Figueiredo and McCarthy 2019; Walden 2019; Hammarström et al. 2020). In addition, insulin, which is secreted from the beta cells of the pancreas in response to rising blood glucose levels, may itself exert anabolic effects irrespective of muscle contraction, e.g., by elevating levels of amino acids, and also plays a role in reducing muscle protein breakdown independent of amino acid availability (Hillier et al. 2000; Abdulla et al. 2016). It seems plausible that combined RT and glucose ingestion provide additive effects on ribosome biogenesis compared to RT alone.

Ribosome biogenesis and content seem to be a prerequisite for skeletal muscle growth, and transcription of ribosomal ribonucleic acid (rRNA) by RNA polymerase I is considered the rate-limiting step in de novo ribosome biogenesis (Moss and Stefanovsky 1995). Multiple proteins and signalling pathways converge to regulate rRNA transcription, including c-Myc and the mammalian target of rapamycin complex 1 (mTORC1) signal-transduction pathway (Kusnadi et al. 2015; West et al. 2016; Tanaka and Tsuneoka 2018; Walden 2019; Mori et al. 2021). First, the general transcription factor c-Myc increases ribosome biogenesis directly through transcriptional control of the upstream binding factor (UBF) (Sanij et al. 2008; Poortinga et al. 2011; West et al. 2016; Mori et al. 2021). Indeed, UBF phosphorylation, which is required for interaction with the ribosomal deoxyribonucleic acid (rDNA) promoter, is increased by RT alone in muscle and also seems to be increased by high-glucose treatment in a mTORC1-dependent manner (rapamycin sensitive) in kidney glomerular epithelial cells (Mariappan et al. 2011).

Second, the mTORC1 pathway receives input from growth factors, hormones, mechanical loading, and nutrients to balance protein synthesis through multiple mechanisms based on cellular energy levels (Hoppe et al. 2009). This contributes to ribosome biogenesis by forming the preinitiation complex (PIC) that marks the initiation of rRNA transcription, as well as through regulation of ribosomal protein translation (Figueiredo and McCarthy 2019; Walden 2019). In addition, mTORC1 and ribosomal protein S6 kinase beta-1 (S6K1) are direct mediators of insulin signalling in skeletal muscle (Hillier et al. 2000). Third, high glucose was shown to lead to chromatin remodelling independent of UBF and mTORC1, which in turn promotes rRNA transcription in cell cultures (Zhai et al. 2012). Together, these mechanistic observations underscore a potential role for glucose in muscle ribosome biogenesis and function in human skeletal muscle, acting in concert with RT to potentiate transcription of ribosomal RNA and increasing translational capacity (Hillier et al. 2000; Hoppe et al. 2009; Zhai et al. 2012; Tanaka and Tsuneoka 2018).

Multiple studies have suggested translational capacity to be as important, if not more important, than translational efficiency for promoting long-term skeletal muscle adaptations to RT (Figueiredo 2019; Hammarström et al. 2020, 2022). While the regulation of translational capacity itself involves activation of c-Myc and UBF, acting to stimulate formation of the PIC through the general transcription factor, as well as through a specific transcription factor facilitating rDNA transcription initiation (Mariappan et al. 2011; Walden 2019), the content of ribosomes, c-Myc and UBF is increased with RT-induced muscle accretion (Hammarström et al. 2020, 2022). Furthermore, Nakada et al. (2016a) found a correlation between rRNA content and rpS6 content in their synergist ablation model on rats. This makes knowledge about factors that regulate and affect ribosome biogenesis key for optimising RT at the individual level.

Therefore, the main purpose of this investigation was to test the hypothesis that glucose supplementation given before and after six RT sessions, conducted over a period of 12 days, will potentiate RT-associated accumulation of markers of ribosomal abundance. Secondly, we aimed to describe the association between changes in total RNA abundance and UBF in human skeletal muscle.

## Materials and methods

All participants gave their written informed consent before study enrolment. The study was approved by the Regional Committee for Medical and Health Research Ethics—South-East Norway (ID nr. 153628), pre-registered at clinicaltrials.gov (Identifier: NCT04545190), and conducted according to the Helsinki Declaration.

### Participants

Sixteen healthy male and female participants (20–33 years, Table 1) were recruited to the study through social media advertisement and word of mouth. The eligibility criteria were non-smoking and moderately trained (i.e. 2–8 RT sessions per 14 days for the last six months). Exclusion criteria were previous injury leading to impaired muscle strength, inability to perform resistance exercise training, symptoms, and a medical record of metabolic disorders including hyperglycaemia. Of the 16 participants that commenced the intervention, three participants dropped out. One due to sickness and inability to resume, while two participants experienced muscular discomfort related to heavy resistance training. Lean mass and body fat % (Table 1) were measured using dual-energy X-ray absorptiometry (DXA, Prodigy Advance PA + 302,047, Lunar, San Francisco, CA, USA) on Day − 1, the last day preceding the RT intervention.

**Table 1:** Participant characteristics: values are means ± SD

Sex	*n*	Age (yrs)	Stature (cm)	Body mass (kg)	Lean mass (kg)	Body Fat (%)
Female	7	24.6 (4.8)	172.1 (5.8)	68.5 (3.5)	49.5 (6.5)	24.6 (8.2)
Male	9	23.7 (1.8)	176.7 (5.0)	78.4 (6.1)	61.1 (4.5)	18.6 (6.5)

### Experimental design

**Fig. 1:**
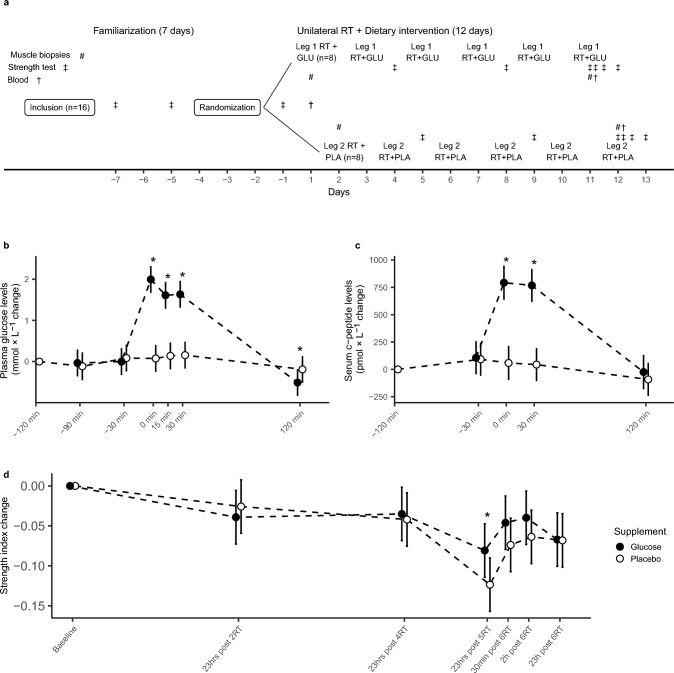
**a** An overview of the experimental design with 12 days of concomitant dietary intervention and resistance training (RT), preceded by 7 days involving familiarization. Between days -7 and -1, participants were familiarized to the RT exercises via 1RM leg press and knee extension testing, and to the strength tests via Humac Norm dynamometer (days − 7 and − 5). Before baseline testing, the participants were randomly allocated to exercise one leg with glucose (GLU) and the other with placebo (PLA), in a unilateral, alternating fashion. Further, non-dominant/dominant + GLU/PLA, and onset with GLU or PLA was also randomized, the figure illustrates an example where the participant was randomized to start RT with GLU. Biopsies were taken from m. vastus lateralis at baseline (Day 1 leg 1, Day 2 leg 2), and after five RT sessions (Day 11 leg 1, Day 12 leg 2). Blood for measuring plasma glucose and serum c-peptide was sampled at baseline (Day 1), and during post-testing (Day 11 leg 1, Day 12 leg 2), via finger draws and venous blood samples. Skeletal muscle strength was measured as peak torque in unilateral isometric and isokinetic (at 60 and 240 degrees per second) knee extension before, multiple times during, and after five and six sessions. **b** and **c** Changes in plasma glucose (b, mmol/L) and serum c-peptide levels (c, pmol/L). Glucose levels in blood were measured via finger draws 120 (− 120), 90 (− 90), and 30 min (− 30) before RT, immediately before RT (0), during RT (15), immediately after RT (30) and 2 h after RT (120). C-peptide levels were measured simultaneously to these finger draws, except for 90 min before and during RT. d) Changes in muscular strength measured as isometric and isokinetic peak torque (60 and 240 d/s) via Humac Norm Dynamometer, conducted at baseline (a: Day − 1), after two and four RT sessions (a: Day 4 and 8 leg 1, Day 5 and 9 leg 2), after five RT sessions/before the 6th session (Day 11 leg 1, Day 12 leg 2), as well as 30 min, 2 h and 23 h after the 6th RT session (a: Day 11/12 leg 1, Day 12/13 leg 2). The index was calculated by normalizing peak torque values to the highest peak torque value at each respective angular velocity, and then summarized and used in change score calculations. Values are presented as changes in estimated marginal means ± 95% confidence intervals (CI). *p < 0.05 between groups. Glucose *n* = 13, placebo *n* = 13

The study was designed as a 12-day double-blinded placebo-controlled simultaneous crossover trial, with an alternating unilateral RT protocol (Fig. 1A). Participants were randomly allocated to exercise one leg with a glucose condition and one leg with a placebo condition (Fig. 1A). One person was exclusively responsible for the randomisation code and supplement distribution, blinding both investigators and participants regarding which leg exercised with glucose/placebo conditions. One bolus of glucose was ingested as 30 g of glucose (Glucosum monohydricum, Merck KGaA, Darmstadt, Germany) and one bolus of placebo was ingested as 100 mg Stevia powder (Steviosa, Soma Nordic AS, Oslo, Norway), containing the natural sweetener erythritol in amounts equivalent to the sweetness of 30 g glucose, mixed with 300 ml Fun Light (Orkla, Oslo, Norway). Hence, the glucose and placebo supplements had identical chemical composition, except for their content of glucose/Stevia. To test whether the boluses truly were masked sufficiently to avoid detection, a blinded taste test was carried out. In this blinded taste test, the participants were given two glasses of glucose mix (75 ml per) and two glasses of the placebo mix (75 ml per), consumed in a randomised order per participant. The participants were instructed to finish one bolus, note their guess for its content, and move on to the next glass. On average, the participants had a score of 2 points (2.08 ± 1.24) out of 4 possible. To ensure equal conditions during training sessions and strength testing, participants exercised and tested at the same time of day, ± 1 h with the same supervisor on pairwise consecutive days (i.e. on days 1–2, 3–4, etc.). To further standardise this, participants also recorded and repeated their daily macronutrient intake (protein, fat, carbohydrate) and total calories on pairwise consecutive days.

All participants completed six RT sessions with glucose and six with placebo, allowing a within-subjects analysis of the effects of glucose ingestion before and after RT. Data from the first five RT sessions was used to investigate main outcome measures (total RNA, rRNA and protein) and leg muscle strength, whereas data from the sixth RT session was used to explore secondary outcomes (muscular recovery, plasma glucose and serum c-peptide levels). Participants were asked to avoid resistance- or high-intensity training of the legs from Day − 7 (Fig. 1A) and onwards, until completion of the intervention and post-testing, to ensure the reliability of pre-intervention strength data and minimise interference from external exercise sources.

### Dietary intervention

The dietary intervention spanned the whole day, divided into three periods: I) From awakening until 2.5 h (hrs) after RT, II) from 2.5 h after RT until 22:00 h, and III) from 22:00 h until awakening. During period I, participants ingested protein and glucose *or* protein and placebo only. Glucose/placebo was ingested three times in period I: 30 min before RT (30 g vs. 0 g glucose), immediately before RT (30 g vs. 0 g glucose), and immediately after RT (30 g vs. 0 g glucose). Whey Protein Isolate (Proteinfabrikken, Stokke, Norway) was ingested 2 h before RT and immediately after RT, in boluses of 25 g mixed with 150 ml water. In the afternoon (18:00–19:00 h, period II) participants ingested glucose or placebo (3 × 30 g vs. 3 × 0 g glucose) opposite to the supplement they received during RT, to ensure a balanced daily intake of glucose. Apart from this, participants ingested a self-chosen diet during period II. Further, participants were asked not to use any other supplements such as additional protein and/or creatine, and to register all food/drink consumption in MyFitnessPal or similar applications. The self-chosen diet was repeated on pairwise consecutive days, to ensure similar premises for resistance training responses between conditions. During period III (22:00–07:00 h), participants remained in an overnight fasted state. The daily onset of the dietary intervention (i.e., first ingestion of protein supplement) varied between participants, from 06:00 to 09:00 h to allow multiple participants to complete the protocol simultaneously. During resistance training sessions, participants were free to ingest water ad libitum.

### Assessment of muscular strength

**Fig. 2:**
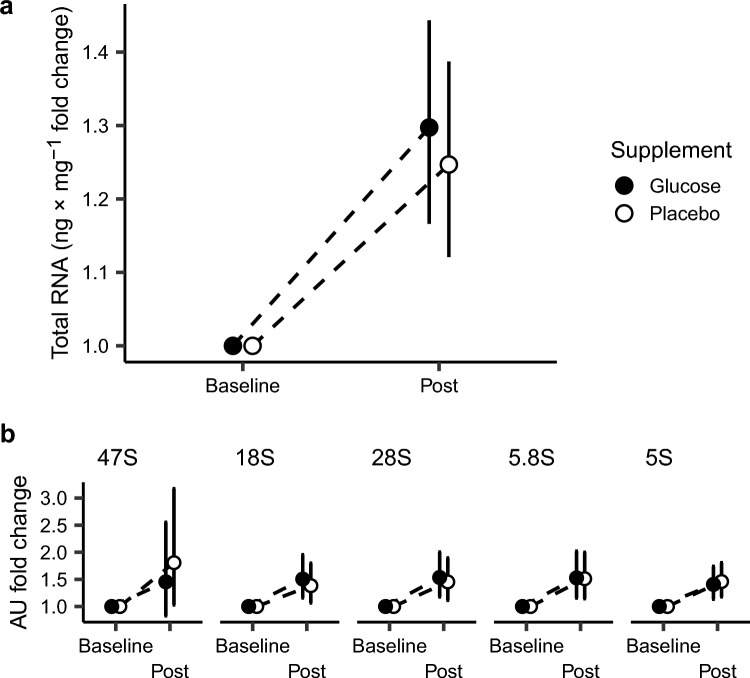
Changes in total RNA and ribosomal RNA with Glucose and Placebo conditions. **a** Total RNA, **b** 47S pre-rRNA, 18S rRNA, 28S rRNA, 5.8S rRNA, 5S rRNA. Baseline = Day 1 leg 1/Day 2 leg 2, Post = Day 11 leg 1, Day 12 leg 2. Total RNA and rRNA were analysed in duplicates, with two duplicates per biopsy (two muscle tissue pieces per time point), and normalized to ng x mg wet muscle weight for total RNA and external reference gene (Lambda) for rRNA. Total RNA and rRNA changes were calculated as log-fold change score per mg wet muscle weight. Mean change scores of the duplicates were calculated and transformed to the log scale before modelling, then reverse-transformed for figure visualisation. Values are estimated marginal means fold change per leg per supplement ± 95% CI. Glucose *n* = 13, placebo *n* = 13

Muscle strength tests were performed before (Fig. 1A, Days -7 and -5, and -1, both legs) and during the intervention (Fig. 1A, on days 4 and 8 for leg 1, and days 5 and 9 for leg two), after session 5 and after finalization of the intervention (Fig. 1A, on days 11/12 for leg 1 and days 12/13 for leg 2). Maximal isometric and isokinetic knee extension torque was measured with a Humac Norm Dynamometer (CSMi, Stoughton, Massachusetts, USA). Individual positions were recorded and standardized from pre-intervention tests (Fig. 1A, days − 7 and − 5). Isokinetic peak torque was measured concentrically from 90 to 0 degrees knee angle (extended knee was set to 0 degrees) at angular velocities of 60 and 240 degrees per second, 2 × 3 repetitions each, with the first set of each exercise as a sub-maximal warm-up. Isometric knee extensor peak torque was measured at a knee angle of 60 degrees, for a maximum of 10 s and two repetitions per test. The isometric tests were ended when the participants reached a plateau or peak torque development decreased, which on average occurred between 2 and 4 s into the test. During days 4, 5, 8 and 9 (Fig. 1D, days 4 and 5 = Post 2RT, days 8 and 9 = Post 4RT), knee extension torque tests were conducted one hour before RT on the leg performing RT the previous day. During days 11 and 12, these tests were performed four times: (I) 45 min before the last RT session (Fig. 1D, Post 5RT), (II) 30 min after the last RT session (Fig. 1D, 30 min post 6RT), (III) 2 h after the last RT session (Figs. 1D, 2 h post 6RT), and (IV) 23 h after the last RT session (Fig. 1D, 23 h post 6RT). Test I on day 12/13 included testing of both legs, representing 23 h post-RT session test of one leg and post-session 5 test of the other leg. The highest peak torque values from the respective angular velocities and time points were summarized in an index. The index was calculated by dividing the average peak torque value by the highest observed peak torque value per angular velocity and summarizing this new index per angular velocity to a mean strength index.

Assessment of unilateral one repetition maximum (1RM) leg press and knee extension was conducted in the familiarisation phase prior to the intervention (Fig. 1, Days − 7 and − 5). The participants performed a general warm-up with 10 min of cycling on an indoor exercise bicycle. A protocol consisting of 1 × 10, 1 × 6 and 1 × 3 repetitions with a load equivalent to ~ 50–75% of assumed maximal repetitions, was used as a specific warm-up before each of the tests. All positions were controlled and recorded at the first 1RM test and reproduced during the RT sessions. Maximal leg press strength was defined as the maximal load lifted in a controlled fashion, starting from a knee angle of 90 degrees. To find a reproducible 90° knee angle for each participant, centimetre markings on the side panels of the leg press machine were used to record where to find 90° for each separate leg and participant. Attempts where participants did not reach 90° during the eccentric phase, were not approved. Maximal knee extension strength testing followed the same specific warm-up as the maximal leg press test and was defined as the maximal load lifted in a controlled fashion, reaching full extension of the knee joint. Attempts with exaggerated hip movement or beneath full extension were not approved. Two minutes of rest were given during the specific warm-up and three minutes of rest were given between 1RM attempts.

### Resistance training protocol

Resistance training consisted of three sets of unilateral leg presses and three sets of unilateral knee extensions, with an exercise intensity of 10 repetitions maximum (10RM). As a general warm-up, the participants cycled on an indoor exercise bicycle for 5–10 min. In addition, before the respective exercises, two 10-repetition warm-up sets were completed at ~ 50% and ~ 70% of 10RM. To ensure adequate exercise stimulation throughout the intervention, the exercise load was increased the following set if the participants lifted more than 12 repetitions, as a progressive loading strategy. If the participants lifted fewer than 8 repetitions per set, the load was reduced in the following set. The resting time between working sets was 2 min. For safety and standardisation purposes, all sessions were monitored by trained personnel. Lastly, training volume (load and repetitions) was logged for every session.

### Sampling of muscle tissue and blood

Muscle biopsies were sampled from m. vastus lateralis using well-established procedures (Hammarström et al. 2020). Briefly, muscle biopsy sampling was performed under local anaesthesia (Xylocaine, 10 mg ml^−1^ with adrenaline 5 μg ml^−1^, AstraZeneca AS, Oslo, Norway) using a 12-gauge needle (Universal Plus, Mermaid Medical AS, Stenløse, Denmark), operated with a spring-loaded biopsy gun (Bard Magnum, Bard, Rud, Norway). After the biopsy sampling, muscle tissue was divided into two aliquots for determination of total RNA/expression of rRNA and two aliquots for protein content measurement. Aliquots were snap-frozen in isopentane (− 80 °C) and stored at − 80 °C until further analyses. Muscle biopsies were collected at four time points: I/II) Before the intervention (Fig. 1A, 2 h before training, Day 1 = leg 1, Day 2 = leg 2), and III/IV) approximately 22 h after the fifth RT session, two hours before the sixth RT session (Fig. 1A, Day 11 = leg 1, Day 12 = leg 2). At each time point, two samples were taken from the same incision. To standardize this procedure, all individual participants had biopsies taken at the same time of day, in an overnight fasted state.

To measure blood glucose levels with and without glucose ingestion/training, capillary blood was collected from finger draws on days with biopsy sampling. One capillary blood sample was collected on day 1 (Fig. 1A) to serve as a baseline. On days 11 and 12 (Fig. 1A), capillary blood samples were collected seven times: I) Immediately before protein ingestion (07:00 h) II) 45 min after protein ingestion (07:45 h) III) 1.5 h after protein ingestion (08:30 h, i.e., immediately before glucose/placebo intake), IV) 2 h after protein ingestion (09:00 h, i.e., immediately before training), IV) in the middle of RT (~ 09:15 h), V) immediately after training (~ 09:30 h), and VI) 2 h after completion of training (~ 11:30 h). Capillary blood samples were analysed with in-house equipment (BIOSEN C-Line, EKF diagnostic GmbH, Barleben). Venous blood samples were collected from the antecubital vein, coinciding with the capillary samples except 45 min after protein ingestion and in the middle of the RT session, to analyse endocrine variables.

### Total RNA extraction and real-time reverse transcription polymerase chain reaction

Two muscle biopsy aliquots were used for total RNA extraction per leg, resulting in a total of eight RNA samples per participant. Total RNA was extracted using TRIzol with muscle tissue homogenised using 0.5 mm RNase-free Zirconium beads (~ 50 ul; Next Advanced, Averill Park, NY, USA) and mechanical agitation (Bullet Blender, Next Advanced, Averill Park, NY, USA). Chloroform (Sigma-Aldrich, Oslo, Norway) was used for phase separation, and the RNA pellet was precipitated with isopropanol (VWR International, Oslo, Norway). To enable analysis of target gene expression per unit tissue weight (Ellefsen et al. 2008, 2014), an exogenous RNA control (Lambda, λ polyA External Standard Kit, Takara Bio Inc., Shiga, Japan) was added at a fixed amount to each sample (0.04 ng ml^−1^ of TRIzol reagent). For assessment of RNA content and purity, RNA was eluted in TE buffer (1:2) and assessed via spectrophotometry. All samples had a 260 nm to 280 nm ratio > 1.9. The RNA stock was stored at − 80 °C until further analyses. Before quantitative analyses of total RNA, samples with known loss of RNA during extraction (*n* = 9) or a deviation from the observed RNA to muscle tissue weight relationship larger than 3 $\times$ residual SD while accounting for training status (*n* = 1) were removed from the data set. Total RNA was normalised to wet muscle weight and log transformed before statistical analyses.

Five hundred ng of RNA was reverse transcribed using Super Script IV Reverse Transcriptase (Invitrogen, Oslo, Norway), according to the manufacturer’s instructions using anchored oligo-dT and random hexamer primers (Thermo Scientific, Oslo, Norway). All samples were reverse transcribed and diluted to 1:50 before quantitative real-time polymerase chain reaction (qPCR). qPCR reactions were run over 40 cycles (3 s 95 °C denaturing and 30 s 60 °C annealing) on a fast-cycling real-time detection system (Applied Biosystems 7500 fast Real-Time PCR Systems, Life Technologies AS), with a total reaction volume of 10 µl consisting of 2 µl of complementary DNA (cDNA), gene-specific primers (0.5 µM final concentration) and a commercial master mix (2X SYBR Select Master Mix, Applied Biosystems, Life Technologies AS, Oslo, Norway). An overview of the primers can be found in Table 2. Raw fluorescence data was modelled with a best-fit sigmoidal model using the qPCR package (Ritz and Spiess 2008) written for R (R Core Team 2020; Hammarström et al. 2020). qPCR data was normalised to wet muscle weight using the external reference gene Lambda (Ellefsen et al. 2008, 2014) and analysed on the log scale on a target-by-target basis.

**Table 2:** Primer sequences: values of Ct are means ± SD

Gene	Sequence (forward—reverse)	Ct mean (SD)	E
18S rRNA	5′-TGCATGGCCGTTCTTAGTTG-3′ 5′-AACGCCACTTGTCCCTCTAAG-3′	9.73 (0.768)	1.82
28S rRNA	5′-TGACGCGATGTGATTTCTGC-3′ 5′-TAGATGACGAGGCATTTGGC-3′	11.0 (0.968)	1.88
5.8S rRNA	5′-ACTCTTAGCGGTGGATCACTC-3′ 5′-GTGTCGATGATCAATGTGTCCTG-3′	15.8 (0.747)	1.81
5S rRNA	5’-TACGGCCATACCACCCTGAAC-3′ 5’-GGTCTCCCATCCAAGTACTAACC-3’	18.4 (0.639)	1.83
47 s rRNA	5´-CTGTCGCTGGAGAGGTTGG-3′ 5′- GGACGCGCGAGAGAACAG-3´	26.1 (1.90)	1.81
Lambda F2R2	5′-AAGACGACGCGAAATTCAGC-3′ 5′- TGGCATTCGCATCAAAGGAG-3′	23.2 (1.50)	2.02
Lambda F3R3	5′-TCGCGGCGTTTGATGTATTG-3′ 5′- TGACGCAGACCTTTTCCATG-3′	23.8 (0.890)	1.81

*rRNA* ribosomal RNA, E = primer efficiency. Average cycle thresholds (Ct) and priming efficiencies were calculated from all qPCR reactions

### Protein extraction and immunoblotting

Total protein was extracted using the Minute Total Protein Extraction Kit for Muscles (Invent Biotechnology), according to the manufacturer’s protocol, optimised for our lab. Wet muscle was freeze-dried for 24 h and dissected before extraction. The tissue was homogenised with a plastic rod in 80 mg protein extraction powder (Invent Biotechnology) and 100 ul ice-cold cell lysis buffer (Denaturing Buffer, Invent Biotechnology), and centrifuged at 19 000 g for 1 min. The supernatant was divided into aliquots to run samples in duplicates, and total protein concentrations were determined in a 1:10 dilution (Pierce Detergent Compatible Bradford Assay Reagent, Thermo Fisher Scientific, Oslo, Norway). The protein samples were diluted to a concentration of 1.5 µg µl^−1^ with lysis buffer and 4X Laemmli sample buffer (Bio-Rad Laboratories AB, Oslo, Norway) containing 2-mercaptoethanol. All protein samples were incubated at 95 °C and stored at − 20 °C until further analysis. The protein samples (20.25 µg total protein) were separated at 250 V on 4–20% Tris–Glycine gels (Bio-Rad Laboratories) for 50 min and then transferred to PVDF membranes with wet transfer at 300 mA for 3 h. Both gel electrophoresis and protein transfer were performed at 4 °C. Following the wet transfer, membranes were stained with a reversible total protein stain (Thermo Fisher Scientific) and then blocked for 1 h at room temperature with a blocking buffer of Tris-buffered saline (TBS; 20 mM Tris, 150 mM NaCl) with 5% non-fat dry milk and 0.1% Tween-20. Primary and secondary antibodies were purchased from Santa Cruz Biotechnology (Texas, USA): UBF, UBF F-9, sc-13125; rpS6, Ribosomal protein S6 C-8, sc-74459; and Thermo Fisher Scientific (Oslo, Norway): c-Myc, 9E10; goat anti-mouse (for c-Myc), goat anti-mouse IgG1 (y1) horseradish peroxidase conjugate; and anti-mouse (anti-mouse IgG1 horseradish peroxidase conjugate). Antibodies were diluted in blocking buffer to concentrations corresponding to 1:500 (UBF and rpS6, primary), 1:2000 (c-Myc, primary), 1:5000 (c-Myc, secondary), and 1:25,000 (UBF and rpS6, secondary).

Membranes were incubated overnight with primary antibodies and for 1 h with secondary antibodies. Between blocking and primary antibody staining, membranes were washed for 5 min, between primary and secondary staining, and after secondary staining, membranes were washed for 3 × 5 min with TBS-Tween (TBS; 20 mM Tris, 150 mM NaCl, 0.1% Tween). Following the last wash, membranes were incubated for 5 min with enhanced chemiluminescent substrate (ECL, SuperSignal West Femto Maximum Sensitivity Substrate, Thermo Fisher Scientific). Membrane blocking, secondary antibody incubation, washing and ECL incubation were performed at room temperature. Primary antibody incubation was performed at 4 °C. Chemiluminescence signals were quantified using Image Studio Lite (LI-COR Biotechnology, Lincoln, NE, USA), and total protein content was quantified using ImageJ (Rueden et al. 2017), where total protein content was defined as mean grey value of the whole well with between-well values subtracted as background. A pooled sample was used as a control on each gel to allow for between-gel comparisons and quantified protein signals were subsequently normalized to the pooled control sample and total protein.

### Statistics and data analysis

A priori power calculations showed that 20 participants would grant a statistical power of 80% (*α* = 0.05), accounting for an expected dropout of 20%. This power calculation was based on an assumption that the effects of glucose ingestion on total RNA accumulation and rRNA expression may equate to the effects of increasing RT volume from low to moderate (Hammarström et al. 2020). Total RNA, protein and qPCR data were analysed by mixed-effects models with fixed effects included as *supplement* $\times$ time. To decrease the risk of Type I errors, random effects were selected from step-wise elimination of terms from the most complex structure (random slopes for time and supplement and their interaction) to less complex. The most complex random effect structure that converged was chosen as the final model (Matuschek et al. 2017). Plasma glucose, serum c-peptide, training volume, and the strength index were analyzed by multiple time-point log-fold change score comparisons, using a mixed-effects model with baseline values, time and supplement, and the interaction between time and supplement as fixed effects. These data only supported a random intercept per participant. The linear mixed-effects models were fitted with the lmer function from the lme4 package using the lmerTest package to procure p-values (Satterthwaite’s method for approximating degrees of freedom) (Bates et al. 2014), written for R (R Core Team 2020). Log-transformed values were expressed as fold changes in visualisations. Descriptive data are presented as mean and standard deviation (SD). Inferential statistics are presented as means with 95% confidence intervals and *p*-values unless otherwise stated. *p* < 0.05 was considered statistically significant.

## Results

Glucose ingestion before and after RT sessions did not lead to a higher mean change of total training session volume, with a mean increase of 17% in both the glucose condition (pre, 5262 ± 1799 kg; post, 6319 ± 2256 kg, *p* > 0.05) and the placebo condition (pre, 5351 ± 1615 kg; post, 6438 ± 2092, *p* > 0.05) from session 1 to session 6, respectively. There were no differences in mean macronutrient intake (protein, fat, carbohydrates) or total calorie intake between glucose and placebo on pairwise consecutive days (*p* > 0.05 for all, raw data and table available in GitHub repository).

Glucose ingestion before and after RT led to increases in plasma glucose levels compared to baseline by 38% immediately before RT (Fig. 1B, 0 min, 2.05 ± 0.73 mmol/L), by 31% during RT (Fig. 1B, 15 min, 1.75 ± 1.44 mmol/L) and by 32% immediately after RT (Fig. 1B, 30 min, 1.62 ± 1.10 mmol/L, all *p* < 0.001), with no changes being observed in the placebo condition (Fig. 1B, 0 min, 0.09 ± 0.3 mmol/L; 15 min, 0.16 ± 0.35 mmol/L; 30 min, 0.18 ± 0.39 mmol/L, all *p* > 0.05). Compared to the placebo condition, ingestion of glucose increased plasma glucose levels by 36% immediately before RT (Fig. 1B, 0 min), by 27% during RT (Fig. 1B, 15 min) and by 28% immediately after RT (Fig. 1B, 30 min, all *p* < 0.001). Two hours after the RT session, glucose ingestion was associated with 12% lower plasma glucose levels compared to baseline, and 8% lower compared to placebo (Fig. 1B, 270 min, *p* = 0.029).

Glucose ingestion before and after RT led to increases in levels of c-peptide compared to baseline, by 95% immediately before (Fig. 1C, 0 min, 796 ± 376.0 pmol/L) and 87% after RT (Fig. 1C, 30 min, 793 ± 581.0 pmol/L, both *p* < 0.001), with no changes observed with the placebo condition (Fig. 1C, 0 min, 63.7 ± 71.0 pmol/L; 30 min, 53.9 ± 134.0 pmol/L; both *p* > 0.05). Compared to the placebo condition, ingestion of glucose increased levels of c-peptide by 85% immediately before (Fig. 1C, 0 min) and 85% after RT (Fig. 1C, 30 min; both *p* < 0.001).

In general, glucose ingestion before and after RT sessions did not improve skeletal muscle recovery compared to placebo throughout the intervention, neither the rested state (Fig. 1D, 23 h after exercise, Post 2RT, *p* = 0.514; Post 4RT, *p* = 0.735), nor acutely after the sixth/final RT-session (30 min post 6RT, *p* = 0.178; 2 h post 6RT, *p* = 0.245) or in the rested state after the sixth RT session (23 h post 6RT, *p* = 0.96). In contrast to this, glucose ingestion was associated with a 7% less reduction in muscle strength after the fifth session compared to placebo (Fig. 1D, *p*  = 0.039).

In knee extension torque, both RT with glucose and placebo led to significantly reduced muscle strength after the fifth session compared to baseline, by 11 and 18% respectively (Fig. 1D, Post 5RT, *p* = 0.000). Comparisons of the acute data gathered from after five sessions until and including 23 h after the sixth session showed an average increase in muscle strength of 5–9% from RT with glucose and placebo 30 min after the sixth RT session (Fig. 1D, 30 min post 6RT, *p* = 0.01) and two hours after the sixth RT session (Figs. 1D, 2 h post 6RT, *p* = 0.004). Twenty-three hours after the last (sixth) RT session, muscle strength was unchanged compared to after the fifth RT session (Fig. 1D, 23 h post 6RT, *p* = 0.117). Table 3 shows the mean change in absolute peak torque values per condition and angular velocity.

**Table 3:** Peak torque: Mean peak torque per condition and angular velocity measured at the different times during the intervention

Condition	Velocity	Baseline	Post 2RT	Post 4RT	Post 5RT	Post 6RT#1	Post 6RT#2	Post 6RT#3
Knee-extension peak torque (Nm)								
Glucose	240° sec^−1^	123.8 (28.8)	127.6 (25.6)	127.2 (29.4)	118.0 (32.7)	118.9 (28.6)	121.3 (30.9)	123.0 (31.5)
Placebo	240° sec^−1^	124.6 (23.3)	128.9 (27.1)	129.7 (28.9)	117.9 (35.6)	118.7 (29.7)	119.7 (30.2)	126.5 (30.8)
Glucose	60° sec^−1^	193.2 (42.3)	201.7 (34.3)	200.2 (35.9)	177.8 (45.3)^*^	182.3 (35.9)	186.6 (42.8)	186.9 (43.8)
Placebo	60° sec^−1^	198.3 (30.5)	201.7 (30.8)	199.5 (38.3)	168.5 (46.7)	171.9 (42.3)	170.2 (42.6)	187.2 (38.1)
Glucose	0° sec^−1^	269.1 (49.1)	277.9 (45.8)	286.2 (54.4)	272.2 (52.2)	264.1 (53.6)	279.1 (53.7)	280.0 (62.4)
Placebo	0° sec^−1^	259.8 (41.6)	286.9 (48.4)	281.5 (51.0)	261.8 (65.2)	251.9 (53.9)	268.6 (45.6)	277.7 (51.2)

Baseline = before exercise, Post 2RT = 23 h after two training sessions, Post 4RT = 23 h after four training sessions, Post RT5 = 23 h after five training sessions, Post 6RT#1 = 30 min after the sixth training session, Post 6RT#2 = 2 h after the sixth training session, Post 6RT#3 = 23 h after the sixth training session. 240º sec^−1^ = 240 degrees per second angular velocity (isokinetic), 60º sec-1 = 60 degrees per second angular velocity (isokinetic), 0° sec-1 = 0 degrees per second angular velocity (isometric). Values are reported as mean ± SD. * = significant difference between glucose and placebo

### Markers of ribosome biogenesis

#### Total RNA and ribosomal RNA

The five-session-RT intervention led to on average ~ 20–27% increases in total RNA (glucose, 263 ± 50 ng/mg^−1^; placebo, 210 ± 121 ng/mg^−1^) and ~ 25–57% increases in rRNA per unit muscle weight (47S, 0.253 ± 1.27 and 0.576 ± 0.677; 18S, 0.336 ± 0.460 and 0.271 ± 0.470; 28S, 0.314 ± 0.504 and 0.311 ± 0.582; 5.8S, 0.388 ± 0.576 and 0.322 ± 0.520; 5S, 0.305 ± 0.608 and 0.292 ± 0.432; arbitrary units for glucose and placebo respectively) (Fig. 2). However, RT with glucose did not induce more pronounced accumulation of total RNA compared to RT with placebo (Fig. 2A, mean difference 7.6%, [− 7.2, 24.9], *p* = 0.337) or rRNA (Fig. 2B, 47S, 37.9%, [− 28.4, 131.6], *p* = 0.400; 18S, − 7.6%, [− 34.0, 29.8], *p* = 0.652; 28S, -2.5%, [− 37.7, 53.2], *p* = 0.915; 5.8S, − 7.7%, [9.8, 98.0], *p* = 0.644; 5S, − 0.4%, [− 31.1, 44.2], *p* = 0.982).

#### Protein

**Fig. 3:**
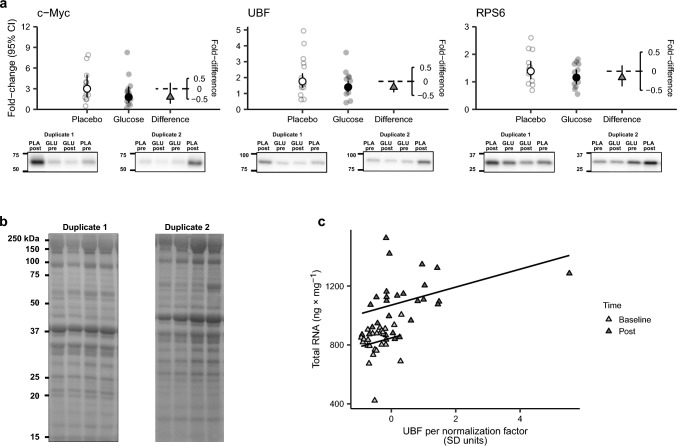
Changes in c-Myc, UBF and RPS6 protein content from pre- to post-training in Placebo and Glucose conditions together with differences between conditions (second axis in (**a**). Representative western blots of the respective proteins are shown under each panel together with total protein stains in (**b**). Protein samples were analysed in two duplicates per biopsy per time point, loaded on separate gels in an inverted order as exemplified by the duplicates (1 and 2 in **a** and **b**). Values are estimated fold change per condition with 95% CI, Glucose *n* = 13 and placebo *n* = 13. A linear relationship was shown between total RNA (ng x mg) and UBF levels (SD units) while controlling for time. Total RNA was normalized by wet muscle weight, and UBF was normalized by a pooled sample used on each gel

The five-session-RT intervention led to increased abundances of all measured proteins, both in the glucose and in the placebo condition (Fig. 3A). RT with glucose resulted in lowered estimates of c-Myc, UBF and rpS6 levels compared to placebo (being − 40, − 21 and − 17% lower compared to placebo, respectively), without reaching statistical significance (Fig. 3A, p = 0.094–0.292). Baseline and trained-state total RNA levels showed a linear relationship with UBF abundances; a $\sim$ 14% increase in total RNA corresponded to 1 SD unit increase in UBF (Fig. 3c, *p*  = 0.0002).

## Discussion

The main findings of the present study were that heavy resistance training with glucose did not affect markers of ribosome biogenesis compared to RT with placebo, measured as total RNA, rRNA, and protein involved in rDNA transcription initiation. Similarly, RT with glucose did not affect markers of skeletal muscle functionality compared to placebo, such as muscle strength and recovery, and total training session volume. Towards the end of the intervention, RT with glucose led to less reduction in muscle strength compared to placebo, which may point to an accumulated effect of the glucose condition. As by design, the levels of plasma glucose and serum c-peptide were significantly higher before, during and after the glucose condition RT sessions compared to placebo, and there were no differences in daily macronutrient intake between conditions on consecutive days, suggesting that the study was adequately designed to elucidate the biological and functional effects of the glucose condition. These findings suggest that the previously observed significance of glucose exposure for rDNA transcription initiation in in vitro studies (Mariappan et al. 2011; Tanaka et al. 2015) is not translatable to acute effects in resistance exercised human skeletal muscle in vivo, with a design like the present study.

The observations of the present study from human skeletal muscle cells do not support previous indications of high glucose exposure on rDNA transcription initiation in non-human and/or non-skeletal muscle cells (Mariappan et al. 2011; Zhai et al. 2012; Tanaka et al. 2015). A UBF-dependent augmentation of ribosome biogenesis did not seem to be induced by glucose ingestion in the present study, as compared to in mice glomerular epithelial cells (Mariappan et al. 2011) and in human breast cancer cells (Tanaka et al. 2015). Neither do our findings indicate that insulin per se, at least at physiological levels, potentiates accumulation of total RNA through p70S6K stimulation as observed with hyperinsulinemia in human skeletal muscle (Hillier et al. 2000). Increases in markers of ribosome biogenesis such as 47S pre-rRNA and mature rRNA are expected to occur after a single session of RT (Figueiredo et al. 2016), as well as after a short period of RT (Hammarström et al. 2020, 2022). Therefore, in the present study, it was expected that any benefits of ingesting glucose compared to placebo with RT would be measurable after five training sessions, either due to glucose-induced stimulation of energy-sensitive pathways such as mTORC1, PIH1, extracellular signal-regulated kinase 1/2 (ERK1/2), AMP-dependent protein kinase (AMPK) or Sirtuin 1 (SIRT1) (Mariappan et al. 2011; Zhai et al. 2012; Kim et al. 2013; Tanaka et al. 2015). Despite previously reported upregulation in PIC assembly due to high-glucose mediated mTORC1, ERK1/2 and PIH1 or low-glucose mediated AMPK and SIRT1 activation (Hoppe et al. 2009; Mariappan et al. 2011; Zhai et al. 2012; Kim et al. 2013; Tanaka et al. 2015), the present study displayed no signs of such effects of glucose vs. placebo conditions.

Importantly, previous studies investigated high vs. low glucose conditions (Mariappan et al. 2011), or high glucose vs. glucose starvation (Hoppe et al. 2009; Tanaka et al. 2015), while the present study aimed to compare the high glucose condition to a placebo condition (stevia), with a matched daily macronutrient and energy intake. Therefore, the comparison made in the present study was high plasma glucose levels vs. normal plasma glucose levels, to investigate the effect of glucose per se and not intracellular energy status. Thus, while glucose ingestion presumably is important for supplying energy for growth-inducing processes such as ribosome biogenesis (Kusnadi et al. 2015; Tanaka and Tsuneoka 2018; Figueiredo and McCarthy 2019) there is no apparent effect of ingesting added glucose per se on markers of ribosome biogenesis, during 12 days of heavy-load RT. Further, previous studies have used cell cultures from yeast (Zhai et al. 2012), rodents (Hoppe et al. 2009; Mariappan et al. 2011) or human breast cancer cells (Tanaka et al. 2015) and are, as such, not directly comparable to human skeletal muscle cells. Nevertheless, resistance training irrespective of condition yielded a robust accumulation of total RNA and expression of rRNA, in line with previous observations for heavy-load RT (Hammarström et al. 2020, 2022).

In the present study, despite not measuring the activity in central pathways mediating anabolic signalling (mTORC1, ERK1/2), analyses of the downstream target UBF and the ribosomal protein S6 (rpS6), as well as the general transcription factor c-Myc were performed. These analyses further supported observations from the RNA data as there was no difference between the glucose and placebo conditions. UBF has previously been described as a master regulator of rDNA transcription in vitro (Russell and Zomerdijk 2005; Kusnadi et al. 2015; Figueiredo and McCarthy 2019), while rpS6 previously correlated with 18S and 28S rRNA content and is proposed as a valid and reliable means to measure ribosome biogenesis (Chaillou et al. 2012; Nakada et al. 2016b). Lastly, c-Myc has previously been described as a potent regulator of ribosome biogenesis, independent of mTORC1, and a direct regulator of UBF (Poortinga et al. 2011; West et al. 2016; Mori et al. 2021). Hence, it seems quite reasonable to observe similar changes in these three proteins. The linear relationship found exclusively between UBF content and total RNA levels, and not between total RNA and c-Myc or rpS6, supports a specific role for UBF in regulating ribosomal content in human skeletal muscle. This is in line with recent observations in human skeletal muscle following a period of RT (Hammarström et al. 2022). The observed RT-induced increase in levels of UBF, c-Myc and rpS6 is itself in line with responses seen in cell cultures and synergist ablation models (Mariappan et al. 2011; Walden et al. 2012).

As in the biological data, combined glucose ingestion and RT did not exert measurable effects on muscular strength throughout the intervention compared to placebo. In general, the skeletal muscle performance index, which was used as a proxy marker for muscular recovery, decreased similarly from baseline to after the intervention in both conditions. There was one exception to this however, as glucose ingestion was associated with a lower reduction in muscular strength after five RT sessions compared to placebo, which may point towards a beneficial accumulated effect where the heavy-load RT gradually fatigued the participants but glucose ingestion counteracted this response (Mul et al. 2015; Tanaka and Tsuneoka 2018). Therefore, we cannot rule out potential long-term benefits of ingesting glucose in connection with heavy-load resistance training, which would require a longer intervention period than that of the present study. Having noted this, glucose did not improve muscular performance/recovery acutely following one RT session compared to placebo, measured 30 min, 2 h and 23 h after the sixth/final training session. As such, the potential accumulated effect observed after five RT sessions did not extend to acute effects measured after RT session six.

The decrease in muscular performance observed over the course of the intervention might be explained by the biphasic recovery pattern, as described by Raastad and Hallén (2000), where the participants experienced a rapid recovery during the initial 11 h after exercise, followed by a levelling off or drop until 22 h after exercise. Herein, inflammation and phagocytic activity were proposed to be involved in the performance drop between 11 and 22 h (Raastad and Hallén 2000). Indeed, this pattern seems quite similar to what was observed in the present study, with a rapid recovery at 30 min and 2 h after the sixth RT session and a drop at 23 h after the sixth session. Further, muscle strength testing during the intervention was conducted 23 h after RT, meaning that the biphasic recovery may have also influenced these tests. However, this neither explains the difference observed after five RT sessions between conditions, nor the drop in muscle strength from after the fourth RT session to after the fifth RT session. A possible explanation could be that exercising without glucose ingestion may have caused more lower cellular energy substrate availability compared to exercising with glucose, as glucose is the preferred energy source during strenuous exercise (Mul et al. 2015), thus increasing performance with glucose compared to placebo. Notably, training volume data displayed that the total training session volume was not different on pairwise consecutive days, i.e. no difference between days 1 and 2, days 3 and 4 and so on. Hence, there were no differences in mechanical loading to induce greater fatigue between conditions. Arguably, an increased energy availability via glucose ingestion during RT may induce less acute fatigue on the exercised skeletal muscle, and perhaps less performance reduction, compared to placebo during RT (Westerblad et al. 1998; Kent-Braun 1999). Unfortunately, we did not conduct measurements of markers of metabolic stress such as inorganic phosphate, H + , Mg2 + and the ADP/ATP ratio (Westerblad et al. 1998; Kent-Braun 1999). Therefore, discussing the potential effect of differences in metabolic stress between conditions would only be speculation, however probable.

### Limitations and strengths

The present study was designed specifically to investigate the acute biological and functional effects of ingesting glucose compared to placebo, with unilateral training and testing in a crossover design. This design allowed for high-resolution analyses of within-participant comparisons of the two treatments and hence removing biological diversity between individuals as a confounding factor (MacInnis et al. 2017). Further, to ensure that the legs were exercised under the same conditions (apart from glucose/placebo during exercise), macronutrients, the time of day and the test/training personnel were standardised for each participant on pairwise consecutive days (Halperin et al. 2015). Every day, the participants showed up in an overnight fasted state and ingested either protein and glucose, or protein and placebo before and after exercise. Taken together, the clear difference between the glucose and placebo conditions in plasma glucose and serum c-peptide, along with the aforementioned standardisations enabled high-resolution analyses of the effect of ingestion glucose on total- and specific RNA levels and proteins, as well as muscular performance, within-participant.

The present study also had a few limitations. Firstly, the sample size was smaller than expected and planned for. Initially the minimum limit of 16 participants, according to the a priori power calculation, was met. However, three dropped out during the intervention. The crossover design (along with all its standardisations) likely still contributed to the validity and reliability of the analyses, though these dropouts possibly left our statistical analyses slightly underpowered. In addition, we did not keep detailed information on each participant's training history prior to enrolment in the study, other than being within our definition (between two and eight RT sessions per 14 days for the last six months). Indeed, this leaves room for variation between participants in terms of training status at baseline, which should be controlled for with the within-participants design (MacInnis et al. 2017). Furthermore, the intramuscular glycogen stores were not measured, hence it cannot be determined whether the glucose ingestion increased intramuscular glucose. Though it may be a reasonable assumption that the glucose ingestion in this design did lead to increased intramuscular glucose and thus energy levels, it cannot be excluded that the participants’ intramuscular glycogen stores were topped up from the previous day, and as such, ingesting more glucose had no further benefit. Importantly though, our main hypothesis was centred around extracellular signalling and the effects of elevated plasma glucose/insulin on rRNA synthesis, and not intracellular energy stores. Nevertheless, even if the skeletal muscle of the leg exercising with the glucose condition took up the extra glucose, it did not seem to affect any of our main outcome measures.

## Conclusion

In conclusion, ingestion of glucose immediately before and after five heavy-load resistance training sessions conducted over 12 days did not augment accumulation of ribosomal RNA, in moderately trained young adults compared to ingestion of placebo. Glucose ingestion did not affect muscular performance throughout the study, nor did it affect muscular performance measured 30 min, 2 h or 23 h after the last session. Glucose ingestion was associated with a lower reduction in muscular performance 23 h after the fifth training session, and we can therefore not rule out a possible accumulated effect of ingesting glucose compared to placebo on recovery. There was a relationship between baseline and trained state data of total RNA and UBF levels. This supports a key role for UBF in ribosome biogenesis in human skeletal muscle following resistance training. Future investigations should focus on the accumulated long-term effects of simultaneous glucose ingestion and RT on RT-related muscular adaptations, as well as include analysis of intramuscular glycogen storage.

## Data Availability

The datasets generated during and analysed during the current study are available in the “ribose-paper” repository; https://github.com/Kristianlian/ribose-paper

## Abbreviations

AMP: Adenosine monophosphate
AMPK: Adenosine monophosphate-dependent protein kinase
ATP: Adenosine triphosphate
cDNA: Complementary deoxyribonucleic acid
CI: Confidence intervals
c-Myc: Cellular myelocytomatosis oncogene
Ct: Cycle threshold
DXA: Dual-energy x-ray absorptiometry
ECL: Enhanced chemiluminescence
ERK1/2: Extracellular signal-regulated kinase 1/2
GLU: Glucose
mTORC1: Mammalian target of rapamycin complex 1
PIC: Preinitiation complex
PLA: Placebo
qPCR: Quantitative polymerase chain reaction
rDNA: Ribosomal deoxyribonucleic acid
RM: Repetition maximum
rRNA: Ribosomal ribonucleic acid
RT: Resistance training
S6K1: Ribosomal protein S6 kinase beta-1
SD: Standard deviance
SIRT1: Sirtuin 1
TBS: Tris-buffered saline
UBF: Upstream binding factor
